# On‐site genetic analysis for species identification using lab‐on‐a‐chip

**DOI:** 10.1002/ece3.7053

**Published:** 2021-01-29

**Authors:** Ryan Wimbles, Louise M. Melling, Bradley Cain, Naomi Davies, Jason Doherty, Bridget Johnson, Kirsty J. Shaw

**Affiliations:** ^1^ Department of Natural Sciences Manchester Metropolitan University Manchester UK; ^2^ Knowsley Safari Prescot UK

**Keywords:** conservation genetics, identification, lab‐on‐a‐chip, microfluidics, rhinoceros

## Abstract

This paper presents a microfluidic device capable of performing genetic analysis on dung samples to identify White Rhinoceros (*Ceratotherium simum*). The development of a microfluidic device, which can be used in the field, offers a portable and cost‐effective solution for DNA analysis and species identification to aid conservation efforts. Optimization of the DNA extraction processes produced equivalent yields compared to conventional kit‐based methods within just 5 minutes. The use of a color‐changing loop‐mediated isothermal amplification reaction for simultaneous detection of the cytochrome B sequence of *C. simum* enabled positive results to be obtained within as little as 30 minutes. Field testing was performed at Knowsley Safari to demonstrate real‐world applicability of the microfluidic device for testing of biological samples.

## Graphical abstract

This paper presents a microfluidic device capable of performing genetic analysis on dung samples to identify White Rhinoceros (*Ceratotherium simum*), which offers a portable and cost‐effective solution for DNA analysis and species identification to aid conservation efforts. Field testing was performed at Knowsley Safari to demonstrate real‐world applicability of the microfluidic device for testing of biological samples, with results in as little as 30 minutes.

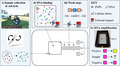

## 1 INTRODUCTION

Microfluidics describes the use of systems which enable the manipulation of small amounts of fluids in channels in the micron range (Whitesides, 2006). Originally conceived from microanalytical methods, such as high‐pressure liquid chromatography (HPLC), the field of microfluidics has rapidly expanded and led to significant benefits in biological research. The ability to conduct work on a miniaturized scale offers a number of advantages over conventional laboratory‐based methods including small reagent and sample requirements, low cost, faster reaction times, and a smaller footprint, all of which are beneficial in producing portable systems (Whitesides, 2006).

Integrated systems capable of “sample in‐answer out” genetic analysis of biological samples have previously been demonstrated, whereby biological samples are added to a microfluidic device and undergo integrated processing, usually consisting of nucleic acid extraction, amplification, separation, and detection (Liu & Mathies, 2009; Park et al., 2014). The vast wealth of literature is also reviewed in a number of more tailored articles on point‐of‐care testing for detection of infectious diseases (Zhang et al., 2017), bacterial pathogens (Lui et al., 2009), forensic analysis (Bruijns et al., 2016), and cancer diagnostics (Newport et al., 2006). Such systems would also be advantageous in conservation and species management settings, for example, where field‐based testing could overcome problems with securing export permits for biological samples, or offering the potential for *in situ* genetic testing in locations where conventional laboratories may not be available.

Despite this, the use of microfluidic devices for genetic analysis in conservation has so far proven limited to single downstream analysis steps, lacking essential prior nucleic acid extraction and amplification, which are still performed using conventional means. DNA sequencing using MinION nanopore sequencers (Oxford Nanopore Technologies) has been demonstrated in the Ecuadorian Chocó rainforest for barcoding of reptile specimens (Pomerantz et al., 2018). Microfluidic single nucleotide polymorphism (SNP) arrays have been applied to analysis of scat samples from wolverine (*Gulo gulo*) (Ekblom et al., 2018), gray wolves, European wildcats, and brown bears (Von Thaden et al., 2017). The use of microfluidic capillary electrophoresis chips has been shown in identifying illegal wildlife trade through restriction fragment length polymorphism (RFLP) analysis, for example, the identification of Malayan box turtle (*Cuora amboinensis*) in traditional Chinese medicines (Asing et al., 2016) and Macaque monkey (*Macaca fascicularis* sp.) in processed foods (Rashid et al., 2015). A full work flow from extraction to identification has yet to have been demonstrated in samples obtained from natural populations. The development of such a system would be extremely valuable to wildlife forensics, conservation, ecology, and taxonomy.

Biological sample collection from animals, particularly those in the wild, is most easily performed through noninvasive methods. Dung samples in particular are extremely valuable as animals regularly defecate, the samples are relatively easy to locate, and the process of collection requires little expertise or expense (Taberlet et al., 1999). However, the use of dung samples for genetic analysis presents challenges due to the heterogeneous nature of the samples, the presence of inhibitors, low target analyte concentrations, and the potential for degraded DNA (Fernando et al., 2003). To date, the analysis of human stool samples on microfluidic devices has mainly focussed on watery samples taken from patients with diarrhea or highly diluted samples (5%–10% fecal samples) to identify infectious agents (Bunyakul et al., 2015; Fronczek et al., 2014; Phaneuf et al., 2018; Ye et al., 2018). More recently, Zhao and colleagues have used acoustic streaming to liquefy human stool samples on a microfluidic device, followed by filtering out of large debris using a micropillar array (Zhao et al., 2019). However, dung samples from herbivores present more of a challenge due to the presence of large amounts of fibrous plant material, and the presence of inhibitors, such as polysaccharides. The use of solid‐phase extraction techniques is beneficial in these scenarios as they facilitate physical separation of the nucleic acids (Fernando et al., 2003), with the added advantage of enabling preconcentration of the nucleic acids which is beneficial when dealing with low target analyte concentrations (Kashkary et al., 2012). We have previously shown how using immiscible filtration assisted by surface tension (IFAST) can be successfully utilized as an example of microfluidic solid‐phase DNA extraction for the detection and analysis of *Helicobacter pylori* in human stool samples (Mosley et al., 2016); however, these techniques have never been applied to nonhuman samples.

In many conservation settings, whether it be zoological institutions or in natural habitats, resources can be limited and therefore the advantages afforded by microfluidic devices can be beneficial. Here, we demonstrate a microfluidic device which is capable of processing animal dung samples, from DNA extraction through to loop‐mediated isothermal amplification (LAMP), to produce a “yes/no” result for species identification. Proof of concept was demonstrated through identification of *Ceratotherium simum*. *C. simum* is a near threatened species that are susceptible in the wild due to poaching for their horns, with 1,054 reported killed in 2016 in South Africa alone (WorldWildlifeFund, 2017). The microfluidic device also incorporates controls to ensure validity of the results. Optimization of the DNA extraction and LAMP steps is demonstrated, alongside field testing of the integrated system.

## 2 MATERIALS AND METHODS

### 2.1 Microfluidic device fabrication

**Figure 1:**
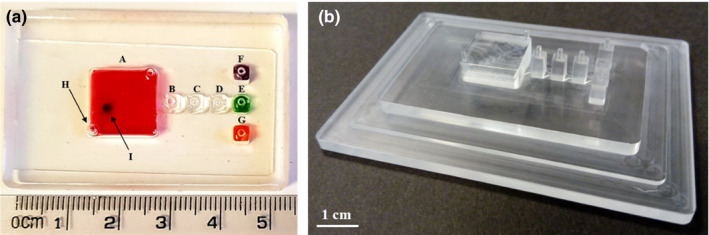
(a) Photograph of the microfluidic device with chambers filled with food dye for easy visualization, containing the following: (A) sample chamber, containing superparamagnetic particles (PMPs) as indicated by I; (B–D) wash chambers; (E) DNA amplification chamber; (F) positive control chamber; and (G) negative control chamber. Each chamber contains 1 or 2 access holes (1 mm diameter) as shown by H as an example; (b) photograph of the PMMA mold showing negative features of the above microfluidic device design

The microfluidic devices used in this experiment were 7‐chamber polymer‐based devices comprising of 5 interconnected chambers for sample analysis (Figure 1a, chambers A–E). The large sample chamber (13 mm wide, 13 mm long, 3 mm deep) was interconnected to four smaller wash and DNA amplification chambers (3 mm wide, 3 mm long, 3 mm deep) via trapezoidal microfluidic gates (3 mm down to 500 µm wide, 1.5 mm long, 250 µm deep). Two independent small chambers are used to contain positive and negative controls (Figure 1a, chambers F & G). The devices were manufactured by pouring a polydimethylsiloxane (PDMS) mixture, consisting of PDMS base and PDMS curing agent in a 10:1 ratio (Sylgard 184^®^ Silicone Elastomer Kit, Dow Corning, UK), into molds designed in Solidworks and produced from polymethyl methacrylate (PMMA) using an M7 CNC milling machine (Datron, UK) (Figure 1b) (Ngamsom et al., 2019). The mixture was degassed by placing in a vacuum chamber for 10 minutes before being cured at 60°C for 1 hr. Once set, the PDMS microfluidic devices were removed from the mold and plasma bonded to a plain glass microscope slide (Fisher Scientific, UK), using a Corona SB device (Blackhole Laboratories, France), to facilitate sealing of the chambers.

### 2.2 Sample collection

A total of 30 dung samples were collected on three separate occasions from a crash which included 9 different *Ceratotherium simum* (White rhinoceros) individuals over the sampling period at Knowsley Safari, Merseyside, UK. The pedigree of the individuals is known and included a number of individuals which were wild caught, as well as their offspring which were captive‐bred. Samples were less than 24 hours old and were taken either from inside the Rhino pens, so they could be associated with one of the nine particular rhino, or from middens within the park where multiple rhinos and other ungulates are present. Sterile forceps were used to collect samples from the outside of the dung pile, ensuring that the sample was collected from areas that had not been in contact with other middens or the enclosure floor. Samples were either used immediately for field testing, or stored in 50 ml universal tubes which had been filled 2/3rds of the way with indicating silica gel (Sigma‐Aldrich) to desiccate the dung samples so they could be taken back to the laboratory for optimization experiments.

Dung samples were also collected from *Diceros bicornis* (Black rhinoceros) from Howletts Animal Park, Kent, UK, using the same method described above. These were analyzed alongside commercially available defibrinated *Equus ferus caballus* (horse) blood (TCS Biosciences) for specificity testing experiments. Additionally, lambda (λ) DNA (Thermo Fisher Scientific) was purchased for use as a positive LAMP control.

### 2.3 DNA extraction

#### 2.3.1 Conventional

Dung and liquid blood samples underwent DNA extraction using QIAamp PowerFecal DNA and DNeasy Blood and Tissue Kits (Qiagen, UK), respectively, following the standard manufacturer's protocol. This was done to enable comparison between commercially available DNA extraction methods and the proposed microfluidic methodology.

#### 2.3.2 Microfluidic

The chambers of the microfluidic device were filled with the following reagents, in sequential order, for optimization of the DNA extraction process: Chambers B and D were filled with mineral oil (Fisher Scientific, UK); chamber C was filled with either (a) mineral oil, (b) 5 M guanidine hydrochloride (GuHCl) in 10 mM TE Buffer (10 mM Tris‐HCl, 1 mM EDTA) (Sigma‐Aldrich, UK), or (c) 70% ethanol (Fisher Scientific, UK) for optimization experiments; and chamber E was filled with molecular biology grade water (Fisher Scientific, UK). All optimization experiments were carried out in triplicate. Dung samples were prepared by vortexing 400 mg of desiccated dung in 4 ml of 5 M GuHCl in 10 mM TE buffer to suspend cells in the solution. A 500‐µl aliquot was then injected into the main sample chamber along with 1 µl of superparamagnetic particles (MagneSil PMPs (Promega, UK)). The microfluidic device was then placed in a custom‐made stand and the PMPs moved from chamber A to E using external NeFeB magnets (one 3 mm dia. × 3 mm height on top of one 22 mm dia. × 10 mm height, Magnet Sales, UK).

### 2.4 DNA quantification

#### 2.4.1 Spectrophotometric

DNA quantification was carried out in order to enable optimization of the DNA extraction process from dung samples on the microfluidic device. Extracted DNA samples were analyzed for total DNA concentration and purity using a NanoDrop™ 2000 Spectrophotometer (Thermo Fisher, UK) at 260 and 280 nm. Molecular biology grade water was used as a blank.

#### 2.4.2 Quantitative PCR (qPCR)

As the extracted DNA samples contain DNA from multiple sources (e.g., *C. simum*, diet, bacteria), qPCR was also performed to specifically detect *C. simum* DNA (see “Primer Design” section) and ensure the DNA extraction protocol on the microfluidic device was effective for the target DNA. The following reagents were used: 1x SensiFAST SYBR^®^ No‐ROX (Bioline, UK), 1 µM forward primer (5′‐AAACTAGGCGGCGTACTAGC‐3′), 1 µM reverse primer (5′‐CATTGGCTTAGGGGTCGGAA‐3′), and 5 µl of template DNA in a total reaction volume of 25 µl. Samples were then run on a Stratagene Mx3000P (Agilent Technologies, UK) using the following parameters: initial denaturation of 94°C for 10 minutes, followed by 40 cycles of 94°C for 30 s, 57°C for 30 s, and 72°C for 30 s, with a dissociation curve performed after the final cycle. Negative controls, containing no template DNA, were also run.

### 2.5 DNA amplification & detection

#### 2.5.1 Primer design

Species‐specific primers were designed against the full *C. simum* cytochrome B sequence (GenBank accession number JF718874.1). A nucleotide BLAST was carried out to compare this sequence with the two full *C. simum* mtDNA sequences available on GenBank (accession numbers Y07726.1 and NC_001808.1), and this showed a 100% match in both cases; therefore, this sequence was taken to be representative of the species based on the available data.

PCR primers were designed using Primer3Plus (https://primer3plus.com/cgi-bin/dev/primer3plus.cgi), and generated an expected amplicon size of 110 bp, and were also used in the qPCR experiments for quantification. We performed *in silico* analysis on the PCR primers using Primer‐BLAST against all mammals and this showed 1 potentially unintended match against *Phocoena sinus* ATP synthase subunit‐a. There were 2 mismatches on the forward primer and 4 on the reverse. This is a species of porpoise which we believe is unlikely to ever be cosampled. We also used the UCSC *in silico* PCR tool which showed only products for *C. simum*.

LAMP primers were designed using PrimerExplorer V4 (https://primerexplorer.jp/e/) (Table 1). LAMP specificity testing *in silico* was evaluated using FastPCR which enables linked searching to be carried out on more than a single primer pair (Kalendar et al., 2017). No unintended products were calculated for any other rhinocerotidae evaluated (*Ceratotherium simum cottoni*, *Rhinoceros unicornis, Diceros bicornis, Rhinoceros sondaicus, and Dicerorhinus sumatrensis*).

**Table 1:** LAMP primer sequences for *Ceratotherium simum*

Primer	Sequence (5′–3′)
Forward Inner Primer (FIP)	TGAGTAGGTCAGCTACTAGTAGTCA‐TCCACACATCAAAACAACG
Backward Inner Primer (BIP)	TCACATGAATCGGAGGTCAACC‐GGTGAAGTATAGGATTGATGCTA
Forward Outer Primer (F3)	ACCCTACTTATTATCCCCTTTC
Backward Outer Primer (B3)	GAGGGGTATAAGTAACTAGGATT
Loop Forward (LF)	GCATTGGCTTAGGGGTCGGAATAT
Loop Backward (LB)	GAACACCCGTTCATCATTATTGGC

#### 2.5.2 Polymerase Chain Reaction (PCR)

PCR was used as a means to confirm the species and quality of DNA obtained from samples, prior to use in the LAMP assay. In addition to the primers for *C. simum* described above, species‐specific primers were also purchased for *D. bicornis* (Brown & Houlden, 1999) and *E. ferus caballus* (Tanabe et al., 2007). PCR was performed using the following reagents: 1x Biomix Red (Bioline, UK), 1 µM forward primer, 1 µM reverse primer, and 5 µl of template DNA in a total reaction volume of 25 µl. Samples were then run, in triplicate, on a Q‐cycler 96 (Hain Lifesciences Ltd, UK) using the following parameters: initial denaturation of 94°C for 10 minutes, followed by 40 cycles of 94°C for 30 s, 50°C, 57°C, or 60°C for 30 s for *D. bicornis, C. simum,* and *E. ferus caballus* respectively, and 72°C for 30 s. Negative controls, containing no template DNA, were also run.

#### 2.5.3 Loop‐mediated Isothermal Amplification (LAMP)

LAMP was used as the amplification method for incorporation onto the microfluidic device. For the LAMP reaction, the following reagents were used: 1x WarmStart Colorimetric LAMP solution (NEB, UK), 0.1 µM F3/B3 primers, 0.4 µM LF/LB primers, and 0.8 µM FIP/BIP primers. For the negative control, the same primers were used but with no template, and for the positive control, 10 ng lambda DNA was added in conjunction with LAMP primers specifically designed for lambda DNA targets (Nagamine et al., 2002). Once the extracted DNA was added, the samples were heated to 65°C on a Prime thermal cycler (Techne, UK) and the reaction allowed to proceed for up to 60 minutes.

#### 2.5.4 Detection

All DNA amplification products were run on a 2% (w/v) agarose gel, made up using 1x TBE buffer (0.1M Tris base, 0.1 M boric acid, and 0.02 M diaminoethanetetraacetic acid (EDTA) sodium salt (Fisher Scientific, UK) in distilled water) and stained with Midori Green™ (Geneflow, UK). Samples were mixed with sample loading buffer and loaded alongside HyperLadder™ 50 or 100 bp (Bioline, UK). Following adequate separation, gels were documented using a UV transilluminator (Geneflash Gel Documentation Darkroom, Syngene, UK). In addition, LAMP reactions were observed visually and photographed, with a positive result indicated by a color change from pink to yellow. The photographs were also analyzed using ImageJ (https://imagej.nih.gov/ij/) to provide RGB values.

### 2.6 Integration and field testing

**Figure 2:**
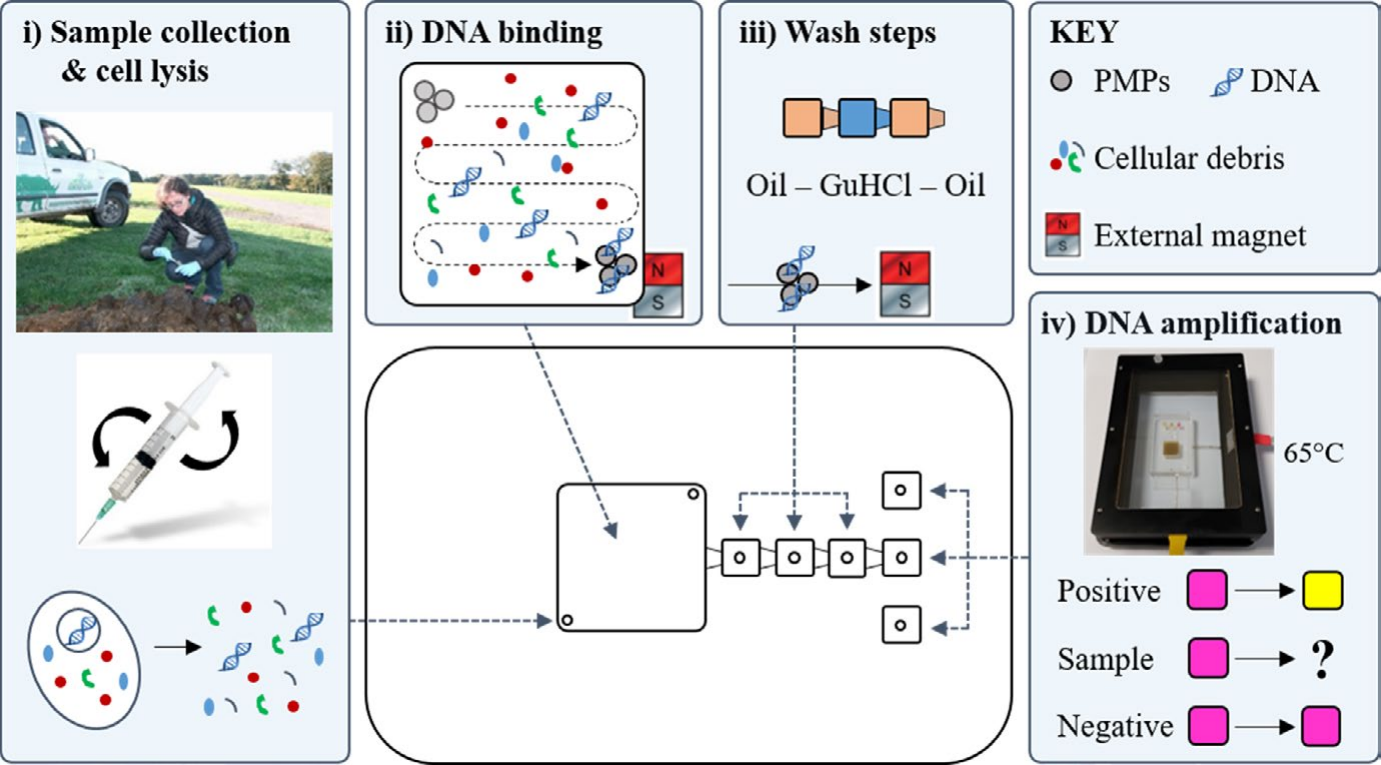
Schematic showing operation of the microfluidic device in the field: (i) dung sample collection with minimal off‐chip preparation and chemical cell lysis; (ii) DNA binding to PMPs; (iii) washing steps; and (iv) DNA amplification using colorimetric LAMP, including positive and negative controls

Once the conditions for DNA extraction and LAMP had been optimized, the molecular biology grade water on the microfluidic device (Figure 1; chamber E) was replaced with LAMP reagents to enable integrated “sample in‐answer out” analysis to be performed. Following confirmation that the integrated procedure was successful in the laboratory, testing was carried out on‐site at Knowsley Safari. Modification of the sample preparation procedure was carried out to facilitate use in the field, without the need for any laboratory equipment, such as a vortex. Dung samples were freshly collected immediately prior to analysis (Figure 2i) and placed in a 2‐mL syringe (BD, UK) containing 1 ml 5 M GuHCl in TE buffer. Once the plunger had been added, the samples were manually inverted 10 times to ensure mixing of the sample and solution, prior to injection into the sample chamber on the microfluidic device through a filter tip to prevent large debris entering the microfluidic device. The microfluidic device was held in a custom‐made stand, and the PMPs in the sample chamber were then mixed with the sample for 2 minutes using an external magnet. The magnet was located directly below the sample chamber and manually moved in a horizontal then vertical serpentine motion (Figure 2ii illustrates DNA binding and an example of horizontal serpentine motion). The magnet was then used to move the PMPs through the three wash chambers (mineral oil, 5M GuHCl in TE buffer, mineral oil) and into the DNA amplification chamber containing LAMP reagents (Figure 2iii). LAMP was performed using a battery‐operated miniaturized heating system (Miniature Incubator (TC‐MIW) and Temperature Controller (TC‐1‐100‐I) (Bioscience Tools, US)) set to 65°C (Figure 2iv). Positive and negative controls were also included on each device simultaneously to enable validation of the results.

## 3 RESULTS

### 3.1 Microfluidic DNA extraction

**Figure 3:**
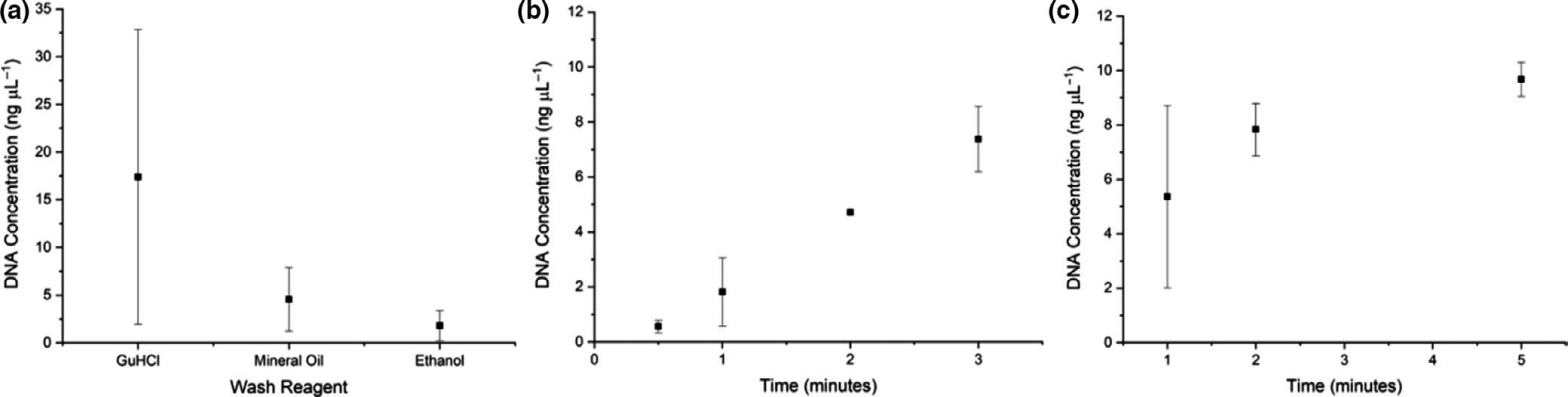
Optimization of the DNA extraction process showing amount of DNA recovered (ng µL^−1^), as measured by qPCR, when (a) wash reagent in chamber C is varied; (b) stir duration in the sample chamber is varied, and (c) elution time is increased. A minimum of three replicates were carried out for all conditions, but in some cases, more experiments were included as the optimum parameters were transferred to the subsequent step (*n* ≥ 3)

Optimization of the DNA extraction processes for dung samples was carried out focussing on evaluation of the following parameters: (a) wash reagent used; (b) stir/incubation time; and (c) elution time. Total DNA yields and purity were assessed using a NanoDrop™, while target specific DNA was quantified using qPCR (see Appendix S1 for example standard curve and melting curve analysis). While chambers B and D contained mineral oil to aid with purification, but also to maintain surface tensions and ensure physical isolation of the wash chambers, the contents of the central wash chamber (C) were varied using either 5 M GuHCl, mineral oil, or 97% ethanol. The results showed that the use of 5 M GuHCl produced higher yields of DNA, although this was not statistically significant (*F* = 2.728, *df* = 2, *p* = .125) (Figure 3a). The amount of time the PMPs were moved around the main sample chamber (A) using the external magnet in order to capture nucleic acids was varied between 0.5 and 3 minutes. When comparing stir times, 0.5 minutes produced significantly lower template DNA compared to the other conditions (*p*'s < .05) (Figure 3b). Finally, the elution time, that is, the amount of time the DNA was left to elute from the PMPs in the final chamber (E), was varied between 1 and 5 minutes. The results showed a positive correlation indicating longer elution times resulting in greater DNA yields (Figure 3c). Analysis of the purity of eluted samples, using 260/280 nm ratios, showed no significant difference at increased elution times (*F* = 0.4473, *df* = 2, *p* = .659).

The optimized protocol was then compared with a conventional QIAamp PowerFecal DNA kit, in triplicate. The results showed no significant differences in DNA extraction efficiency (*t* = 1.769, *df* = 4, *p* = .152) between the two methodologies.

### 3.2 LAMP DNA amplification

#### 3.2.1 Specificity testing

**Figure 4:**
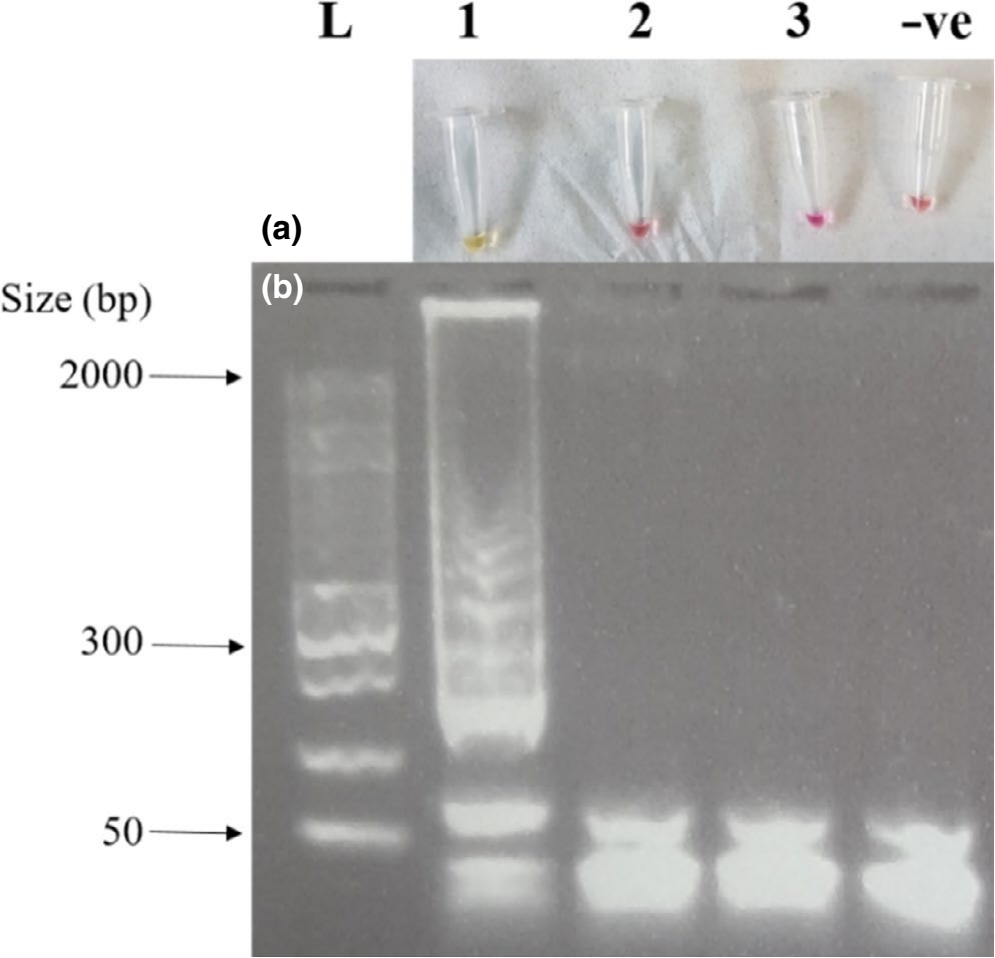
Experimental cross‐species testing was conducted for the LAMP assay: (a) Photograph showing colorimetric results; (b) gel electrophoresis image showing results of specificity testing of LAMP primers where L is DNA size ladder, 1 is *Ceratotherium simum*, 2 is *Diceros bicornis*, 3 is *Equus ferus caballus*, and –ve is the negative control containing no DNA

In addition to the *in silico* specificity testing described in the methodology, experimental evaluation against closely related species identified through phylogenetic analysis (Price & Bininda‐Emonds, 2009) was carried out on those samples which could be physically obtained and included *D. bicornis* and another *Perissodactyl, E. ferus caballus*. Visual examination showed a positive reaction for *C. simum*, and no reaction against *D. bicornis, E. ferus caballus* or for the negative control (Figure 4a). This was then confirmed using gel electrophoresis (Figure 4b). All DNA extracts were also successfully amplified using the polymerase chain reaction with species‐specific primers to ensure DNA of amplifiable quality was present.

#### 3.2.2 Speed of analysis

**Figure 5:**
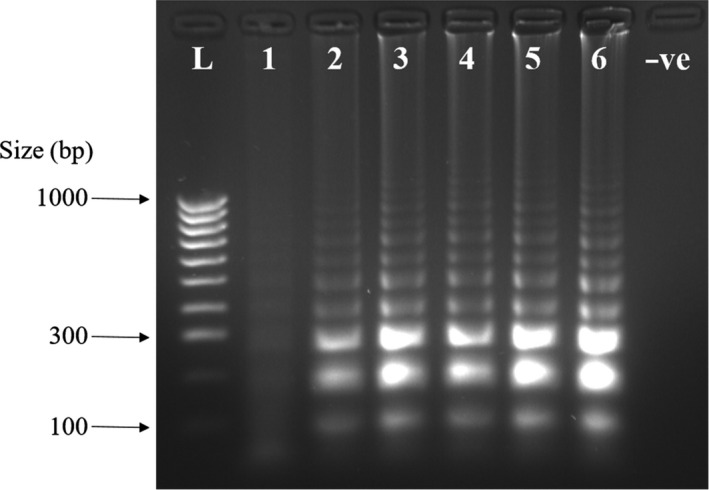
Gel electrophoresis results showing amplification products at various time points, where L is the DNA size ladder, 2–6 are time periods of 30, 45, 60, 90, and 120 minutes, respectively, and –ve is the negative control

The time taken to achieve a positive amplification result using the LAMP primers, with DNA extracted from *C. simum*, was also investigated and showed products from as little as 30 minutes when visualized using gel electrophoresis (Figure 5). Visual analysis of the color change (pink to yellow) was also carried out, and RGB analysis showed statistically significant differences from 45 minutes compared to control samples (*p* < .05).

### 3.3 Field testing of the integrated microfluidic device

**Figure 6:**
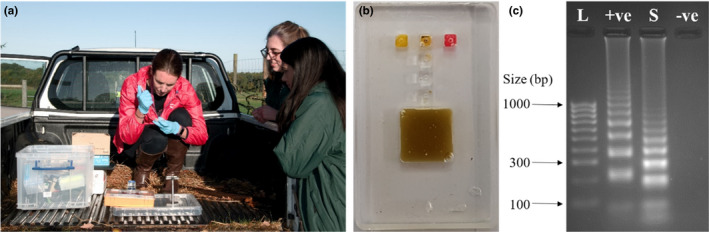
(a) Photograph showing field testing of the microfluidic device at Knowsley Safari, UK, including staff participation; (b) example image of a microfluidic device that was tested in the field following DNA amplification (*n* = 4); and (c) gel electrophoresis image showing results from samples tested in the field and brought back to the laboratory for confirmation analysis, where L is DNA size ladder, +ve is the positive control with lambda DNA, S is the extracted DNA from the dung sample processed on the integrated microfluidic device, and –ve is the negative control with no DNA present

Once optimized, the microfluidic devices were taken to Knowsley Safari for field testing. Fresh dung samples were collected and then analysis took place on the back of a pick‐up truck within the park (Figure 6a). Integrated DNA extraction and amplification were performed, and the on‐site results showed that a visual color change was observed in the dung sample and positive control chambers (Figure 6b). Processed samples were removed from each of the amplification chambers on the device and the results confirmed back in the laboratory by gel electrophoresis (Figure 6c). Knowsley Safari staff, including research and conservation officers and rhino keepers, were able to participate in the use of the microfluidic devices while on‐site (Figure 6a).

## 4 DISCUSSION

### 4.1 Microfluidic DNA extraction

A 5‐chamber microfluidic device design was chosen as the use of three sequential wash chambers has been shown to be effective in extracting DNA of high purity from human stool samples (Mosley et al., 2016). Optimization of the extraction process demonstrated that the use of GuHCl in the central wash chamber resulted in higher DNA yields, most likely as a result of the chaotropic nature of the solution maintaining DNA on the PMPs and preventing loss of DNA within the microfluidic device, compared to the use of alcohol or mineral oil (Tian et al., 2000). Previous studies have shown that nonspecific binding of DNA on untreated PDMS can range from 20 to 23 ng DNA/cm^2^ (Esmerats et al., 2013).The amount of time the sample was incubated with the PMPs, that is, stir time, showed no improvement in the amount of DNA captured between 1 and 5 minutes; therefore, 2 minutes was selected as the stir time as a compromise of speed and consistency (lowest standard deviation). For the elution times, there was a positive correlation between elution time and DNA yield; however, this needs to be balanced with the total reaction time and ensuring practicality in field‐based applications. As a result, an elution time of 2 minutes was chosen, allowing the entire DNA extraction process to be completed within 5 minutes. No significant differences in sample purity upon increased elution times were observed, indicating that potential contaminants had been successfully removed by a combination of physical separation and washing. Comparison of the optimized microfluidic protocol and a conventional kit‐based extraction have shown no significant difference between the two methodologies. The use of the microfluidic device offers advantages in terms of reduction in the overall speed of analysis (<5 minutes compared to approximately 1 hr) and portability for field‐based applications but without a reduction in the amount of DNA recovered.

### 4.2 LAMP DNA Amplification

A species‐specific LAMP reaction has been developed for *C. simum,* which offers a number of advantages over conventional PCR‐based methods of identification. These include simpler operating systems, small DNA template targets, and faster, visual detection (Becherer et al., 2020).

### 4.3 Field Testing of the Integrated Microfluidic Device

Field testing of the microfluidic devices demonstrated that species identification from dung samples could be confirmed using the system within 1 hr, compared with the previously reported DNA sequencing methods carried out in the field which were able to analyze specimens within 24 hr of collection (Pomerantz et al., 2018). Dung samples are added to the device, and a color change, from pink to yellow, is produced if the target species is present. This enables operation by nonspecialist personnel who can visualize the control and sample amplification chambers to check for a reaction, overcoming technical and language barriers. The work presented here demonstrates proof of concept in using such systems within the field, although the easy adaptation of the microfluidic device using alternative LAMP primers opens up possibilities in many areas of conservation where rapid, cost‐effective, portable genetic testing would be beneficial particularly in scenarios where a simple “yes/no” result is required. More broadly, it could benefit the conservation and management of threatened taxa with particular utility in wildlife forensics, for example, the identification of animals in traditional Asian medicines or species confirmation from products seized as part of the illegal wildlife trade, and population monitoring of species with overlapping range whose dung is difficult to reliably identify, for example, similarities between eld's deer (*Rucervus eldii siamensis*) and muntjac (*Muntiacus muntjac*) dung, making field collection of samples more effective.

## CONFLICT OF INTEREST

The authors declare that they have no conflicts of interest to report.

## AUTHOR CONTRIBUTIONS

**Ryan Wimbles:** Formal analysis (equal); investigation (equal). **Louise M. Melling:** Investigation (equal); writing–review and editing (equal). **Bradley Cain:** Formal analysis (equal); writing–review and editing (equal). **Naomi Davies:** Resources (equal); writing–review and editing (equal). **Jason Doherty:** Resources (equal). **Bridget Johnson:** Resources (equal); writing–review and editing (equal). **Kirsty J. Shaw:** Conceptualization (equal); methodology (equal); project administration (equal); supervision (equal); writing–original draft (equal); writing–review and editing (equal).

## Supporting information

Fig S1

## Data Availability

Data sharing is not applicable to this article as no new data were created or analyzed in this study. All experimental data are contained within the manuscript.
