# Supplementary material for: On‐site genetic analysis for species identification using lab‐on‐a‐chip
Source: Ecol Evol. 2021 Jan 29;11(4):1535–43. doi: 10.1002/ece3.7053 (PMC7882957; doi:10.1002/ece3.7053)
Supplement: Supplementary file 1 — Fig S1 [file ECE3-11-1535-s001.docx]

**Appendix**
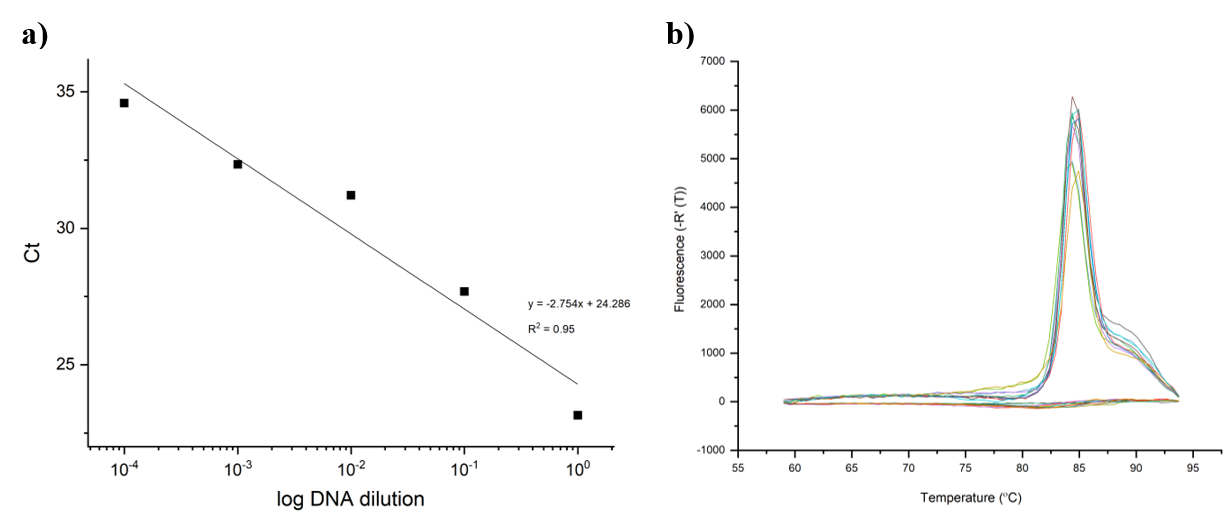


**Figure A1:** a) An example standard curve produced from *C. simum* DNA diluted in a 10 fold dilution series and analysed using qPCR; b) An example of melting curve analysis following qPCR from DNA extracted from *C. simum* dung samples.
